# Childhood obesity governance in Qatar: a policy and legal benchmarking analysis against international child rights and WHO frameworks

**DOI:** 10.1186/s12889-026-28785-0

**Published:** 2026-08-04

**Authors:** Mariam Alisi

**Affiliations:** 1 Department of Social & Policy Sciences, University of Bath, Claverton Down, Bath, BA2 7AY UK; 2 College of General Education, University of Doha for Science and Technology, Al Tarafa, Duhail North, P.O. Box 24449, Doha, Qatar

**Keywords:** Childhood obesity, Qatar, Normative benchmarking, Policy surveillance, Schools, Food marketing, Accountability

## Abstract

**Background:**

Childhood obesity prevention requires coherent national policy packages that address children’s food, physical activity, school, health service, and governance environments. Yet country-level policy claims are often made without auditable verification against primary legal and policy texts. This study assessed the extent to which retrievable Qatari instruments align with the United Nations Convention on the Rights of the Child and World Health Organization benchmarks for childhood obesity prevention and management.

**Methods:**

A single-country, document-based normative benchmarking design was applied. Official Qatari legal, strategic, guideline, and operational instruments were identified through structured searches, domain-guided searches of official repositories and targeted snowball sampling. Fifteen instruments formed the final scored corpus. Provisions were extracted as verbatim excerpts with location pointers, then coded across 12 benchmark-derived domains to assess alignment and the plausibility of delivery. The latter distinguished general or guidance-oriented text from provisions specifying implementation, monitoring, accountability, compliance, or enforcement features. Findings were synthesized descriptively through an instrument-by-domain matrix; no statistical tests were conducted.

**Results:**

Among the 180 coded instrument–domain cells within the retrieved corpus, 34 were aligned under the study-specific framework: five A2 cells, indicating complete alignment with the study-defined minimum, and 29 A1 cells, indicating partial alignment; 146 were not aligned. Alignment was most evident in governance, monitoring, food-environment measures beyond marketing, early-life prevention, and child/adolescent obesity services. Prominent features included multisector governance commitments, surveillance and reporting, dietary and physical-activity guidance, and clinical guidance for childhood obesity management. Within the retrieved and scored corpus, no instrument contained a benchmark-consistent package of child-focused unhealthy-food marketing restrictions. Evidence on school food and physical activity was limited to time-bound operational instruments.

**Conclusions:**

The scored corpus demonstrates several policy foundations but also uneven alignment and variable specificity of delivery. Priorities for further verification and, where gaps are confirmed, for policy development include child-focused food marketing regulation, more comprehensive school measures, and clearer accountability. Findings are limited to retrieved documents and do not assess implementation, enforcement, or health outcomes.

**Supplementary Information:**

The online version contains supplementary material available at https://doi.org/10.1186/s12889-026-28785-0.

## Introduction and literature context

Childhood obesity is increasingly framed as a systems problem rather than a phenomenon reducible to individual choice, because children’s dietary and activity patterns are shaped by commercial practices, institutional settings, and policy environments that determine what is affordable, accessible, and heavily promoted. Global analyses of long-run trends in thinness and obesity underscore that the distribution of childhood and adolescent weight outcomes has shifted in many settings over recent decades, with obesity increasing in numerous countries alongside changing food environments and sedentary exposures [1]. In high-income contexts and rapidly urbanizing settings, this shift often coincides with the strong penetration of energy-dense ultra-processed foods, pervasive marketing, and reductions in routine physical activity, with growing attention to the “commercial determinants of health” as a policy-relevant frame for describing how corporate strategies can shape risk exposures and health equity [1, 2]. This framing is directly relevant to childhood obesity because it shifts analytic attention from downstream counselling to upstream conditions that structure children’s exposure to persuasive marketing, price signals, and default food and activity environments.

A child-rights perspective provides a normative basis for expecting governments to protect children from foreseeable environmental harms, including those mediated by commercial practices. The Convention on the Rights of the Child (CRC) defines the child and articulates State duties relevant to health and its determinants. General Comment No. 15 clarifies the right to the enjoyment of the highest attainable standard of health, and General Comment No. 16 addresses State obligations regarding business-sector impacts on children’s rights [3–5]. These texts do not specify obesity policies in checklist form, but they establish expectations that can be operationalized for benchmarking: protecting the conditions for healthy development, addressing underlying determinants, and regulating third parties when their practices undermine children’s rights [4, 5]. This complements WHO’s governance framing, in which obesity prevention is repeatedly positioned as requiring enabling-environment measures and multisector action rather than relying primarily on downstream clinical services [6–8]. In this study, the child-rights and WHO governance anchors are treated as complementary: child-rights texts provide normative justification and accountability expectations, while WHO instruments specify priority domains and policy design features relevant to enabling environments.

Qatar offers a relevant case for studying how these global benchmarks translate into domestic legal and policy architectures. Publicly available national-level datasets and syntheses indicate that measured child and adolescent overweight/obesity is a documented public health concern in the country, including evidence from Qatar’s school growth monitoring program and secondary reporting in global observatory products [9, 10]. At the same time, as in many jurisdictions, relevant governance content is typically distributed across a combination of binding legal instruments (laws, regulations, and implementing decisions where available) and non-binding instruments (strategies, action plans, guidelines, technical frameworks), which can still be influential in practice but differ in formal enforceability. Accordingly, the analysis explicitly treats “legal force” as an empirical attribute of the domestic corpus and retains non-binding guidance by operationalizing policy intent in a form that can be assessed for its specificity and accountability design features, rather than assuming that non-binding instruments are analytically irrelevant.

Methodologically, a core challenge in obesity governance research is that many reviews either remain at the level of reporting that a strategy exists or focus narrowly on a single policy domain (for example, taxes or school food standards), without assembling an auditable, text-grounded map of what the jurisdiction’s instruments actually say. More recently, policy surveillance and scientific legal mapping have emerged as methodological responses to this problem by treating laws and policies as data: systematically identifying texts, applying transparent interpretations, and coding them into structured datasets that can be updated and compared [11, 12]. This study adopts that logic by constructing a text-retrievable corpus from authoritative repositories, documenting the corpus-construction process, and restricting analytic claims to provisions supported by verifiable primary text.

Another gap is that legal and policy mapping often records whether a measure exists without separately assessing whether the text specifies responsibilities, compliance mechanisms, monitoring, reporting, enforcement, or other delivery-related features. This study, therefore, employs a study-specific dual-coding approach that distinguishes normative alignment from delivery plausibility at the level of provision. Health in All Policies (HiAP) is used only as a supporting governance reference for policy coherence and cross-sector coordination, and the audit trail links coding decisions to verbatim excerpts and location pointers [13, 14].

### Evidence gap addressed by this study

Given the study’s design and the broader literature, the most defensible gap claim is not that Qatar lacks policies, but that publicly available, auditable, provision-level mapping of Qatar’s childhood obesity governance across binding and non-binding instruments remains limited. In addition, even where mapping studies exist in other settings, many primarily document whether policy measures are present, without explicitly separating normative alignment from reviewable delivery-design features. This distinction matters because the existence of a policy statement does not, on its own, demonstrate feasibility or accountability; implementation-focused work on childhood obesity prevention shows that effectiveness depends on policy design, implementation context, and maintenance over time [15], while food-marketing policy reviews show that effects vary by policy scope, design, and implementation [16].

This study advances the literature by providing an auditable, provision-level normative benchmark of Qatar’s childhood obesity governance across binding and non-binding instruments and by integrating normative alignment and delivery plausibility within a single transparent coding framework.

### Study objectives

This single-country study constructs a text-retrievable corpus of Qatar’s binding and non-binding instruments relevant to childhood obesity prevention and management. It benchmarks these instruments against a study-defined global reference set that combines child-rights standards and WHO obesity/NCD governance guidance, without treating programmatic guidance as legally binding. Relevant provisions were coded for normative alignment and delivery plausibility, while child relevance was recorded as a separate descriptive attribute. Indeterminate categories were available when the meaning of the retrieved text did not permit confident scoring. Provision-level results are then synthesized into an instrument-by-domain alignment matrix and a governance appraisal, with claims traceable to evidence pointers and verbatim excerpts rather than to inferences about implementation or impact.

Accordingly, this study asks: To what extent do the retrievable Qatari legal and policy instruments align with international child-rights and WHO benchmarks for childhood obesity governance, and to what extent do aligned provisions contain reviewable delivery-related features?

### Conceptual framework and analytic approach

This study is organized around three complementary pillars: a child-rights framework, which provides the normative basis for protecting children’s health and regulating harmful third-party practices; a study-specific set of WHO childhood-obesity and noncommunicable-disease governance benchmarks, which defines the substantive policy domains assessed; and policy surveillance and legal mapping, which provide the methodological basis for identifying, extracting, and coding domestic legal and policy provisions. Clinical and epidemiological evidence is retained as contextual justification. Systems perspectives, commercial determinants of health, global health law, Health in All Policies, and implementation science are used as supporting concepts rather than as separate analytical frameworks.

### Clinical and epidemiological context

Childhood overweight and obesity are defined using age- and sex-standardized anthropometric measures rather than adult body mass index thresholds. The WHO growth reference for children and adolescents aged 5–19 years classifies overweight as a BMI-for-age greater than one standard deviation above the reference median and obesity as greater than two standard deviations above the reference median, corresponding to adult-equivalent BMI values of 25 and 30 at age 19 [17]. Childhood overweight and obesity are associated with immediate and longer-term health risks, including an increased risk of noncommunicable diseases across the life course, supporting prevention as a public-health priority [18, 19].

Within Qatar, peer-reviewed school growth-monitoring data document changes in schoolchildren’s weight-status distribution across the 2016–2017 and 2019–2020 survey periods [9]. These epidemiological findings do not establish causation, policy failure, or the effectiveness of any particular intervention. Rather, they provide contextual justification for examining whether the retrievable Qatari legal and policy instruments contain provisions relevant to the prevention and management of childhood obesity.

### Pillar 1: child-rights framework

The child-rights framework provides the principal normative foundation for the analysis. Qatar, as a State Party to the Convention on the Rights of the Child (CRC), is subject to a framework concerning children’s health, development, non-discrimination, and protection from preventable harm. The CRC does not provide a checklist of childhood obesity policies, but it establishes principles that can inform the assessment of whether domestic governance arrangements protect the conditions necessary for children’s health and development [3].

General Comment No. 15 on the child’s right to the highest attainable standard of health is particularly relevant because it addresses childhood obesity and children’s exposure to unhealthy foods. It recommends that states reduce children’s exposure to unhealthy foods, regulate the marketing of such foods to children, and control their availability in schools and other settings [4]. This supports treating food environments, marketing practices, and child settings as matters relevant to states’ protective health responsibilities rather than solely to individual or family choice.

General Comment No. 16 addresses state obligations concerning the effects of business-sector activities on children’s rights. Although it is not specific to obesity, it supports examination of food and advertising markets through a child-rights perspective, including regulation, oversight, accountability, and protection from foreseeable commercial harms [5]. Together, General Comments No. 15 and 16 provide the normative justification for examining whether the retrieved instruments address children’s exposure to obesogenic environments and harmful commercial practices.

A global health law perspective supports the relevance of both binding and non-binding governance instruments. Laws and regulations may establish prohibitions, standards, inspection powers, or sanctions, while strategies, guidelines, and action plans may define objectives, assign responsibilities, and outline monitoring arrangements. The latter are not treated as legally binding obligations in this study, but their stated design features remain relevant to understanding the documented policy architecture. Legal force is therefore recorded separately from the substantive alignment and delivery-related characteristics of individual provisions [20, 21].

### Pillar 2: WHO childhood-obesity and NCD governance benchmarks

The WHO benchmark pillar reflects the understanding that childhood obesity is shaped by food systems, marketing practices, schools, health services, physical-activity environments, and other institutional and commercial conditions. Systems and commercial-determinants perspectives help explain how product availability, pricing, promotion, digital marketing, and built environments influence children’s exposures and available choices [22–24]. These perspectives support the relevance of upstream policy action but are not applied as separate analytical frameworks.

The study-specific WHO benchmark set identifies policy domains spanning food and marketing environments, schools, physical activity, early-life prevention, health services, multisector governance, monitoring, and accountability. The principal instruments include the Global Strategy on Diet, Physical Activity and Health; the Global Action Plan for the Prevention and Control of Noncommunicable Diseases and its implementation roadmap; the Global Action Plan on Physical Activity; the Commission on Ending Childhood Obesity report; WHO’s 2010 recommendations and 2023 guideline on food marketing to children; and the WHO Acceleration Plan to Stop Obesity [6–8, 25–28]. The UNICEF–WHO food-marketing toolkit provides additional implementation-oriented guidance relevant to policy development, monitoring, and evaluation [29].

These WHO instruments are used as normative and programmatic benchmarks rather than as legally binding standards. HiAP is retained as a supporting governance reference for intersectoral coordination and policy coherence, not as an independent analytical framework [14]. The WHO Eastern Mediterranean Regional Office obesity-prevention framework and related GCC/MENA materials provide regional context but do not alter the global criteria used for scoring [30–33].

### Pillar 3: policy surveillance and legal mapping

Policy surveillance and legal mapping provide the methodological foundation for treating legal and policy texts as analyzable data. These approaches support systematic instrument identification, transparent inclusion and exclusion decisions, provision-level extraction, structured coding, and preservation of links between coded findings and the underlying primary texts [11, 12, 34].

In this study, these methods are applied to a predefined, retrievable corpus rather than to Qatar’s entire policy landscape. Coding is based on discrete provisions accompanied by source excerpts and location pointers. This approach supports an auditable account of what is demonstrable within the retrieved and scored documents while avoiding inferences about instruments or provisions not included in the scored corpus, actual implementation, enforcement, effectiveness, or population-level outcomes.

Policy surveillance and legal mapping also support the construction of the instrument-by-domain matrix through which provisions are evaluated consistently against predefined benchmark expectations. This methodological pillar, therefore, distinguishes findings derived directly from scored texts from broader conclusions about national policy performance.

### Application to Qatar and the analytic model

In Qatar, governance content on childhood obesity is distributed across binding laws and regulations and non-binding national strategies, sector strategies, guidelines, clinical guidance, and operational instruments. These documents differ in legal force and policy function. National strategies, including the National Health Strategy 2024–2030, provide relevant planning context, while the earlier National Health Strategy 2018–2022 offers continuity in national health planning [35–37]. However, the existence of a strategy or guideline does not, by itself, demonstrate normative alignment, implementation, enforcement, or effectiveness.

The three pillars lead to two related analytical questions. First, the child-rights and WHO benchmark pillars define the substantive expectations against which retrieved Qatari provisions are assessed. Second, policy surveillance and legal mapping determine how those provisions are identified, extracted, coded, and aggregated. The analysis, therefore, examines whether provisions correspond to the predefined benchmark domains and, separately, whether aligned provisions contain reviewable delivery-related features such as assigned responsibility, monitoring, reporting, compliance, enforcement, or accountability mechanisms. The resulting model provides a corpus-bounded, text-based assessment of normative alignment and stated delivery specificity. It does not assess implementation fidelity, enforcement in practice, service coverage, policy effectiveness, or childhood obesity outcomes.

## Methods

### Study design and analytic orientation

A single-country, document-based normative benchmarking design was used to assess the alignment of Qatar’s domestic legal and policy framework for childhood obesity prevention and management with a predefined set of global health and child rights benchmarks. The analysis centers on “policy on the books,” examining the content of retrievable primary texts and assessing whether provisions are textually reviewable for delivery, including clear requirements, designated responsible actors, monitoring and reporting expectations, enforcement mechanisms, and integrity safeguards where explicitly stated. This study does not estimate causal effects on obesity outcomes or assess implementation fidelity. This approach is consistent with policy surveillance and legal epidemiology, which treat laws and policies as codified governance instruments that can be systematically collected, coded, and analyzed [38]. This permits Qatar’s childhood obesity governance architecture to be assessed using retrievable primary texts rather than broad or unverifiable claims about policy presence. The benchmark framework served as the external evaluative reference; no other country or jurisdiction was used as a comparator. Regional documents are used exclusively to contextualize interpretation and salience, without modifying the benchmark set used for scoring. Accordingly, the study is a policy-surveillance and legal-mapping analysis using normative benchmarking; it is not an evaluation of implementation, legal compliance, or comparative national performance.

### Benchmark corpus

The benchmark corpus was organized around two anchors:

- Child-rights anchor: the CRC and authoritative interpretive guidance relevant to child health and determinants, including State duties regarding third-party (business sector) practices.
- Global health governance anchor: WHO instruments framing obesity/NCD risk as an enabling-environment problem requiring multisector policy action across diet, physical activity systems, marketing to children, child settings (including schools), and monitoring/acceleration tools.

Additionally, the WHO Health in All Policies guidance served as a reference for governance appraisal, informing the interpretation of governance design. One regional framework was retained for contextual purposes but was not scored as a global benchmark. The list of benchmark instruments used in the study is provided in Table 1.

**Table 1:** Benchmark instruments used to assess childhood obesity governance and their roles in the benchmarking matrix

	Benchmark instrument	Issuing authority	Year / version	Primary policy focus for benchmarking	Role in this study	Ref.
1	Convention on the Rights of the Child (CRC)	UN General Assembly (treaty)	1989 (in force 1990)	Child rights obligations; defines child; state duties relevant to health and determinants	Core benchmark (alignment)	[3]
2	CRC General Comment No. 15 (Right to health)	UN Committee on the Rights of the Child	2013	Child’s right to health; determinants; state duties relevant to prevention and health systems	Core benchmark (alignment)	[4]
3	CRC General Comment No. 16 (Business sector)	UN Committee on the Rights of the Child	2013	State duties to regulate business impacts on children’s rights (including marketing/food environments)	Core benchmark (alignment)	[5]
4	Global Strategy on Diet, Physical Activity and Health (DPAS)	WHO (endorsed by WHA)	2004 (WHA57.17)	Enabling environments; multisector approach to diet/physical activity risk factors	Core benchmark (alignment)	[7]
5	Global action plan for the prevention and control of NCDs	WHO	2013–2020 (extended to 2030 by WHA72(11) in 2019)	NCD governance; diet/physical inactivity as major risk factors; monitoring/accountability framing	Core benchmark (alignment)	[6, 42]
6	Implementation roadmap 2023–2030 for the Global action plan for the prevention and control of NCDs 2013–2030	WHO	2023–2030	Delivery acceleration + accountability to implement NCD/risk-factor action	Core benchmark (alignment)	[25]
7	Global action plan on physical activity 2018–2030 (GAPPA)	WHO	2018–2030	Systems approach to increasing PA across settings (schools, transport, communities, etc.)	Core benchmark (alignment)	[26]
8	Set of recommendations on the marketing of foods and non-alcoholic beverages to children	WHO (endorsed by WHA63.14)	2010	Restrict marketing of unhealthy foods/non-alcoholic beverages to children	Core benchmark (alignment)	[27]
9	Policies to protect children from the harmful impact of food marketing (WHO guideline)	WHO	2023	Policy features + implementation considerations to protect children from food-marketing harms	Core benchmark (alignment)	[43]
10	Report of the Commission on Ending Childhood Obesity (ECHO)	WHO	2016	Life-course + child-setting recommendations (schools/food env./health services)	Core benchmark (alignment)	[8]
11	WHO Acceleration plan to stop obesity	WHO	2023	Delivery-oriented multisector implementation framing to accelerate obesity prevention action	Core benchmark (alignment)	[28]
12	Health in All Policies: Helsinki statement. Framework for country action	WHO	2014 (statement adopted 2013; framework published 2014)	Governance coherence; intersectoral coordination; policy integration	Governance appraisal reference (not an additional obligation)	[14]
13	Regional framework for action on obesity prevention 2019–2023 (EMRO)	WHO Regional Office for the Eastern Mediterranean (EMRO)	2019–2023	Regional policy domains + progress indicators (Eastern Mediterranean context)	Contextual reference (not scored as global compliance)	[30]

### Translating benchmarks into benchmark-derived domains and coding rules

Benchmarks were operationalized into a domain-based benchmarking matrix (D1–D12), with each domain representing a benchmark-derived category that requires verifiable textual evidence to be coded as aligned. Minimum expectations and decision rules for each domain are specified; the benchmark-derived domains and their anchor instruments are listed in Table 2. Definitions of domains and coding decision rules are provided in the Supporting Information (Table S1).

**Table 2:** Benchmark-derived domains operationalized in the benchmarking matrix and the main anchor instruments used to define minimum expectations in each domain

#	Benchmark domain	Operational meaning in this study (minimum expectation level)	Primary anchor instruments (examples)
1	Child right to health and underlying determinants	Instrument explicitly frames child health protection and links it to underlying determinants relevant to diet and physical activity (e.g., nutrition, healthy development, food/physical activity environments).	CRC (Art. 24); CRC General Comment No. 15; WHO DPAS (2004); WHO ECHO report (2016)
2	Non-discrimination and child equity considerations	Codable equity/non-discrimination features affecting children’s access to prevention and health-supportive environments, where the instrument makes the issue reviewable (e.g., explicit non-discrimination duty, priority groups, access safeguards).	CRC (Arts. 2–3); CRC General Comment No. 15
3	Business sector impacts and protection of policy integrity	State duties to regulate and oversee business-sector impacts on children’s rights and health (including harmful marketing practices), and codable safeguards for transparent management of conflicts of interest where stated.	CRC General Comment No. 16; HiAP Helsinki Statement & Framework; WHO 2010 marketing recommendations; WHO 2023 food-marketing guideline
4	Food marketing restrictions to protect children: foods high in saturated fats, trans-fatty acids, free sugars and/or salt (HFSS)/ “unhealthy” products	Measures that reduce children’s exposure to, and the persuasive power of, unhealthy food marketing (including digital), with codable design features (scope, settings/media, techniques, enforcement/accountability).	WHO 2010 marketing recommendations; WHO 2023 food-marketing guideline; CRC General Comment No. 16
5	School food environment	Codable standards/restrictions/procurement or availability rules for foods/drinks in schools/childcare (where specified), including any compliance/enforcement features.	WHO ECHO report (2016); WHO DPAS (2004)
6	School physical activity environment	Codable requirements/standards enabling physical activity in schools (e.g., physical education (PE)/active school provision, opportunities and supportive settings), where specified.	WHO DPAS (2004); WHO GAPPA (2018–2030); WHO ECHO report (2016)
7	Community/built environment for child physical activity	Codable supports for active living through built environment/transport/recreation/community settings (where specified), including whole-of-society enabling actions.	WHO GAPPA (2018–2030); WHO DPAS (2004)
8	Food environment measures beyond marketing	Codable upstream levers beyond marketing (e.g., policy packages that make healthier choices easier, standards, fiscal/public-sector measures, enabling environments). Captured as present/absent and design features only when the instrument specifies them.	WHO DPAS (2004); WHO Global NCD Action Plan; WHO Acceleration Plan to Stop Obesity (2023); (contextual: WHO EMRO obesity prevention framework 2019–2023)
9	Early life-course prevention (maternal/infant/early childhood)	Codable early-life measures (pregnancy/infancy/early childhood nutrition, healthy growth, breastfeeding-related support where framed), when specified.	CRC (Art. 24); CRC General Comment No. 15; WHO ECHO report (2016)
10	Obesity prevention and management within health services	Codable health-service commitments relevant to children/adolescents (prevention support and/or management/treatment) only to the level specified; do not assume detailed pathways unless stated.	WHO ECHO report (2016); WHO Acceleration Plan to Stop Obesity (2023); (contextual: WHO EMRO obesity prevention framework 2019–2023)
11	Multisector governance and policy coherence (HiAP-type mechanisms)	Codable governance design: mandates, roles, coordination mechanisms, and whole-of-government/whole-of-society structures; not aspiration alone.	HiAP Helsinki Statement & Framework; WHO Global NCD Action Plan; WHO NCD Implementation Roadmap 2023–2030; WHO Acceleration Plan to Stop Obesity (2023)
12	Monitoring, surveillance, evaluation, and reporting	Codable indicator systems, surveillance, evaluation, and reporting/accountability commitments that make implementation reviewable.	WHO Global NCD Action Plan; WHO NCD Implementation Roadmap 2023–2030; CRC General Comment No. 15; HiAP Framework; (contextual: WHO EMRO obesity prevention framework 2019–2023)

Two study-specific ordinal classifications were applied:

- Alignment classification (A-code): captures the degree of correspondence between a coded provision and the study-defined minimum expectation for the relevant benchmark domain. A2 indicates that the provision meets the complete operational definition of the minimum expectation specified in Table S1; A1 indicates relevant but incomplete correspondence; A0 indicates that no qualifying aligned evidence was identified; and A9 indicates that alignment could not be determined confidently from the available text. A2 therefore denotes complete alignment with the study-defined domain minimum and does not imply comprehensive policy adequacy, legal compliance, implementation, or national performance.
- Delivery-plausibility classification (D-code): captures the number of reviewable delivery-related features stated in an aligned provision. D0 indicates no codable delivery-related feature; D1 indicates one feature; D2 indicates two or more distinct features; and D9 indicates that delivery-related specification could not be determined confidently. Delivery was coded only where alignment was present (A1 or A2).

The D2 threshold was adopted as a pragmatic ordinal distinction between an isolated delivery-related marker and a multi-feature textual specification. Relevant features, where explicitly stated, included assigned responsibility, implementation or compliance mechanisms, monitoring or reporting, enforcement, and accountability. The features were not weighted, and no single feature or combination of features was deemed sufficient for successful implementation. D2, therefore, indicates greater textual delivery specification under the study’s operational rules and does not demonstrate implementation capacity, fidelity, effectiveness, or performance.

Illustrative coding example: Q23 states: “The purpose of this guideline is to define the appropriate prevention, diagnosis and management of obesity in children.” This provision was mapped to D10 and coded A2 because it directly met the study-defined minimum expectation for a child-specific health-service commitment covering prevention and management. It was coded D0 because the excerpt did not assign responsibility for implementation or specify monitoring, reporting, compliance, enforcement, or accountability. The reference to healthcare professionals as intended users was not treated as an assignment of implementation responsibility.

#### Development and operationalization of the coding framework

The dual coding framework was developed specifically for this study rather than adopted as a pre-existing validated scoring instrument. Policy surveillance, scientific legal mapping, and legal epidemiology informed the systematic identification, extraction, and coding of legal and policy texts [11, 34]. Implementation science concepts, including CFIR and the implementation-outcomes literature, were used only to support the separate assessment of stated delivery-related features, including assigned responsibility, monitoring, reporting, compliance, enforcement, and accountability [39–41]. The study did not operationalize the full CFIR framework, measure implementation outcomes, or assess actual implementation performance.

The framework was developed by the researcher specifically for this study as a rule-based method to organize and compare heterogeneous provision-level evidence. The 12 benchmark domains and their minimum expectations were derived deductively from the child-rights and WHO instruments listed in Tables 1 and 2. The framework was intended as an analytical organizing structure, not as a psychometrically validated scale or a national policy performance index.

The specific A- and D-code thresholds were defined pragmatically to support a parsimonious and transparent classification of the source texts. No formal external validation exercise was conducted. The categories, therefore, describe the coded content of the retrieved provisions in accordance with the study’s operational rules and should not be interpreted as direct measures of national policy performance or implementation. Supporting Table S1 documents the operational definitions and decision rules used in the analysis.

### Qatar corpus construction, corpus boundary, and instrument classification

#### Search strategy and retrieval documentation

Domestic instruments were identified by one researcher through structured searches, domain-guided searches of official Qatar legal and governmental repositories and the websites of relevant issuing bodies, supplemented by targeted snowball searches of references, titles, and institutional links found in the retrieved documents. The search focused on legal and policy instruments relevant to the 12 benchmark domains. Supporting Table S2 summarizes the official sources and issuing bodies associated with the scored instruments, the broad eligibility and search concepts, the included instrument IDs, and the archived source files. The corpus was finalized on 6 February 2026. The search was designed to construct a transparent and auditable corpus of publicly retrievable primary texts and was not intended to serve as a complete census of Qatar’s policy landscape. After identification, exact titles and instrument numbers were used to confirm official provenance and retrieve stable primary texts.

#### Inclusion/exclusion and scored corpus boundary

Items were eligible if issued by governmental authorities (or constituted official legal texts) and contained provisions plausibly relevant to childhood obesity governance through food environments, marketing exposure, child settings (including schools), physical activity environments, health-system services, surveillance, or cross-sector governance arrangements. Items were excluded if they were non-authoritative, duplicative or superseded, non-normative summaries, outside the child/adolescent scope, insufficiently specific for provision-level coding, or could not be defensibly mapped to the predefined domains.

All alignment, governance, and gap findings are explicitly limited to the scored corpus, which includes instruments with retrievable primary texts and completed provision-level extraction and coding. The scored corpus comprises 15 Qatar instruments (Q1–Q5, Q9, Q11, Q14–Q16, Q20–Q21, Q23, Q25–Q26). An initial identification and screening phase generated an inventory of 26 candidate instruments from Qatar considered plausibly relevant to childhood obesity governance. The scored corpus was subsequently restricted to instruments meeting the study’s inclusion criteria: (i) issued by an authoritative governmental body or constituting an official legal text; (ii) primary text retrievable in a stable, archivable form suitable for audit; and (iii) eligible for provision-level extraction under the benchmark domains and evidence rules. Instruments were excluded if they were out of scope for the child/adolescent benchmarking frame (e.g., adult-only clinical guidance), were non-normative summaries, were duplicates, or were superseded versions for which the operative text could not be confirmed. Application of these criteria resulted in a final corpus of 15 Qatar instruments. The remaining 11 candidates did not meet one or more of the inclusion criteria. The principal exclusion reasons were population-scope mismatch, duplication or supersession, insufficiently specific content for provision-level coding, or no defensible mapping to the predefined domains. The final corpus should therefore be understood as the set of eligible instruments identified, retrieved, and scored under the documented search and evidence rules, rather than as a complete census of all relevant instruments in Qatar. In accordance with scope rules, Q22 (adult obesity clinical guideline) was retained in the project inventory but excluded from scoring because it addressed adult obesity, explicitly excluded persons younger than 18 years, and contained no extractable child-, caregiver-, or household-focused provision eligible for mapping. Although parental weight status and household environments may influence the risk of childhood obesity, downstream family relevance could not be inferred without codable child- or family-specific text. The Qatar instruments retained in the final scored corpus are listed in Table 3.

**Table 3:** Qatar instruments included in the scored corpus

ID	Official title	Issuing body	Instrument type	Legal force class	Status	Period/ year	Child relevance
Q1	Law No. (8) of 1990 on the Regulation of Human Food Control	State of Qatar	Law	L1	In force/current*	1990	Implicit
Q2	Law No. (4) of 2014 amending provisions of Law No. (8) of 1990	State of Qatar	Amending law	L1	In force/current*	2014	Implicit
Q3	Law No. (8) of 2008 on Consumer Protection	State of Qatar	Law	L1	In force/current*	2008	Implicit
Q4	Law No. (25) of 2018 regarding Excise Tax	State of Qatar	Law	L1	In force/current*	2018	Implicit
Q5	Cabinet Decision No. (5) of 2019 issuing the Executive Regulations of the Excise Tax Law	Council of Ministers (Cabinet)	Implementing regulation	L2	In force/current*	2019	Implicit
Q9	Qatar Third National Development Strategy 2024–2030 (NDS3)	National Planning Council	National strategy	P1	Current (active policy cycle)	2024–2030	Mixed (child-related components)
Q11	National Health Strategy 2024–2030	Ministry of Public Health (State of Qatar)	Health sector strategy	P1	Current (active policy cycle)	2024–2030	Mixed (child-setting/child-related components)
Q14	Qatar Dietary Guidelines (booklet)	Ministry of Public Health	Dietary guidelines	G1	Current	2015	Mixed (includes child guidance)
Q15	Qatar Dietary Guidelines for Mother and Child (0–5 years)	Ministry of Public Health	Maternal/child nutrition guidance	G1	Current	Not stated in document	Explicit
Q16	Qatar National Physical Activity Guidelines (QNPAG), 2nd edition	Aspetar	National physical activity guidelines	G1	Current	2021	Explicit
Q20	Primary Health Care Corporation (PHCC) Corporate Strategic Plan 2024–2030	PHCC	Corporate strategy	P2	Current (active policy cycle)	2024–2030	Mixed (includes child-related priorities)
Q21	Hamad Medical Corporation (HMC) Healthcare Strategy 2024–2030	HMC	Corporate strategy	P2	Current (active policy cycle)	2024–2030	Implicit
Q23	National Clinical Guideline: The Management of Obesity in Children	Ministry of Public Health	Clinical guideline	G1	Current	Valid from 25 June 2020	Explicit
Q25	Qualifying food companies to supply food in school canteens for the academic year 2024/2025	Ministry of Education and Higher Education (MOEHE) School Canteens Committee	Pre-qualification notice + conditions/specifications	P3	Time-bounded (AY 2024/2025)	2024/2025	Explicit (school setting)
Q26	Physical Fitness Evaluation for public school students in the State of Qatar for the academic year (2019–2020)	MOEHE – Department of Physical Education	Programme/standards document for student fitness evaluation	P3	Time-bounded (AY 2019–2020)	2019–2020	Explicit (public school students)

All instruments were analyzed using publicly available English-language versions issued or hosted by official governmental or institutional sources. No researcher-generated translation was undertaken

*Abbreviations*: *L1 Law/Act* L2 Executive regulation, *P1* National strategy/action plan, *P2* Sector/corporate strategy, *P3* Ministry administrative/operational instrument, *G1* Guideline/clinical guideline, *MOEHE* Ministry of Education and Higher Education

* For legal instruments, “in force/current” reflects inclusion as current instruments in the study corpus; formal legal status beyond the corpus was not independently verified.

#### Instrument attributes and language of source documents

Each instrument was classified by type and formal legal force class, including binding laws or acts, executive regulations, national strategies or action plans, sector or corporate strategies, ministry administrative or operational instruments, and guidelines or clinical guidelines. Legal force is treated as a formal status attribute, while enforceability is assessed separately at the provision level based solely on explicit textual features, such as penalties, licensing or inspection conditions, and compliance obligations, rather than being inferred from instrument type. Instrument status, period, and child-relevance flags were recorded as metadata for interpretation.

All 15 Qatari instruments included in the scored corpus were analyzed using publicly available English-language versions issued or hosted by the relevant governmental or institutional authorities. No researcher-generated or internal translation was undertaken. The analysis was based on the published English-language texts retrieved for coding and did not involve translation between Arabic and English. A language note is provided beneath Table 3.

### Extraction and coding

#### Unit of analysis and evidence rules

The unit of analysis is the provision, defined as a discrete, codable text unit that includes a requirement, prohibition, standard, institutional arrangement, monitoring obligation, enforcement mechanism, or implementation design feature relevant to one or more benchmark domains. Extraction followed a predefined, strict-evidence rule: each record had to include a location pointer, such as an article, section, or page number where available, and a verbatim excerpt from the primary text. No provision was coded based on inferred intent, secondary summaries, or unverifiable references. When meaning-critical ambiguity could not be resolved from the primary text, the record was excluded from scoring (and documented as excluded) rather than inferred or approximated.

#### Coding fields

Each extracted provision was coded for: domain mapping (D1–D12), benchmark domain link identifier, child-specificity flag (child-explicit / child-implicit / child-setting), policy lever type, provision type, specificity level, Alignment (A-code), and Delivery plausibility (D-code). Cross-cutting governance markers were also coded at the provision level to support governance appraisal and gap typing: accountability assignment (as extractable), monitoring/reporting characterization, enforcement hook characterization, and conflict-of-interest safeguards only when explicitly stated in the excerpt.

#### Coding workflow and quality assurance

Framework development, document searching, screening, extraction, and coding were conducted by a single researcher using a structured Microsoft Excel workbook with predefined fields, controlled value lists, and coding rules. Because only one researcher was involved, formal multi-coder training, blinded independent coding, consensus resolution between reviewers, and inter-rater reliability testing were not undertaken. Coding ambiguities were resolved by consulting the predefined decision rules, the relevant benchmark expectation, and the primary source text. A second-pass quality-assurance review checked provision excerpts, location pointers, domain assignments, A- and D-code values, normalization to controlled value lists, and child-specificity classifications against the primary texts. To support transparency and reproducibility, coding decisions were retained alongside the relevant primary-text excerpts, source locations, decision rules, and quality-assurance records. The master extraction dataset contained 104 provision records; after deduplication, out-of-scope checks, and quality-assurance corrections, 101 provisions were retained for analytic counts.

### Aggregation, matrix construction, and synthesis

Provision-level codes were aggregated conservatively, with traceability preserved to the underlying provision identifiers and excerpts:

- Instrument by domain alignment matrix: For each instrument and domain cell, the cell-level alignment value is determined from the provision rows mapped to that cell. If at least one included provision maps to a cell, the cell-level alignment reflects the highest alignment level observed (A2 over A1). Cells with no mapped provision rows are treated as A0 for matrix completeness; the supporting data retain whether a cell is empty versus populated by A0-scored provisions.
- Instrument by domain delivery summary: For aligned cells, cell-level delivery is summarized conservatively from provision-level D-codes mapped to the cell, with D-codes only when A indicates alignment, as specified in Table S1.
- Domain-level synthesis: For each domain, synthesis summarizes the contributing instruments, provision distributions, alignment-strength patterns, delivery-plausibility patterns, and child-specificity coverage. These summaries inform the main Results, governance appraisal, and gap register. All derived tables and the full provision-level dataset are provided in the Supporting data.

### Governance design appraisal and gaps

Governance design appraisal assessed whether the scored provisions contained reviewable delivery-related features, distinguishing direction-setting text from provisions with more highly specified responsibilities, and identifying monitoring, reporting, compliance, enforcement, or accountability mechanisms. The appraisal used the D-code and the provision-level governance markers and did not assess actual adoption, implementation fidelity, effectiveness, or population-level delivery.

- Coverage gaps: domains with no extractable aligned provisions anywhere in the scored corpus.
- Design-quality gaps are domains where alignment exists but is limited, partial-dominant, time-bound, or not at a higher textual-specification level. This includes low specificity, limited delivery features, weak or unclear monitoring design, limited enforceability, or the absence of integrity safeguards in the extractable text, as defined by the rules in Table S1.

All gap statements are explicitly bounded to the scored corpus and do not claim jurisdiction-wide absence unless verified by corpus expansion.

### Transparency, reproducibility, and availability

Reproducibility is supported through a versioned extraction dataset, verbatim excerpts, location pointers for included provisions, explicit inclusion/exclusion flags with rationales, and retained quality assurance audit sheets. The Supporting Information (Table S1) provides the domain framework and scoring rules. The study uses only publicly available documents and does not involve human participants or identifiable personal data.

**Fig. 1:**
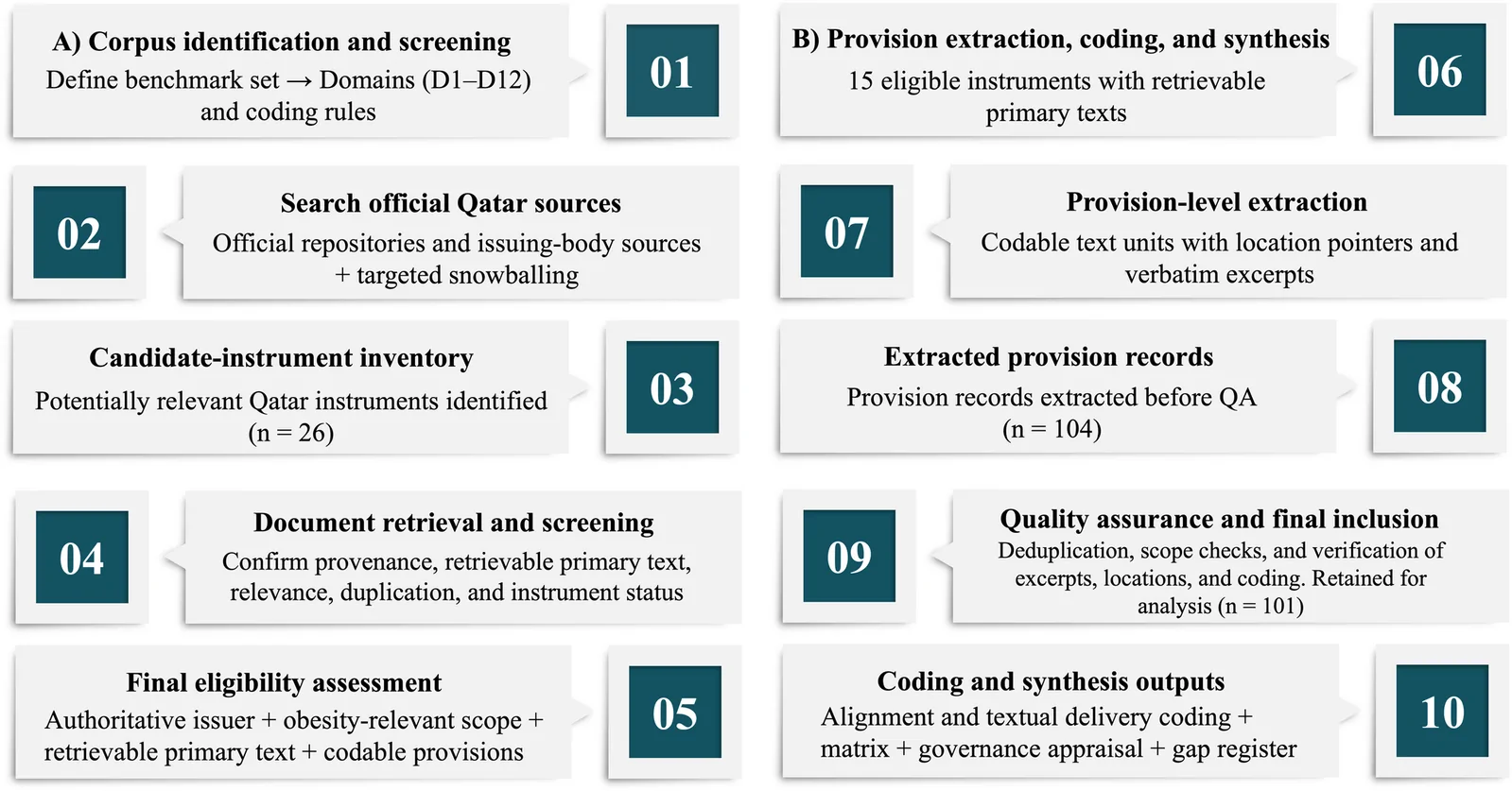
Structured workflow for corpus identification, provision extraction, quality assurance, coding, and synthesis

Figure 1 summarizes the study workflow for corpus identification and scoring (Panel A) and for provision extraction, quality assurance, coding, and synthesis (Panel B), resulting in the alignment matrix and gap register. The figure is presented as a structured workflow diagram and is not intended to indicate that the study was conducted as a PRISMA-compliant systematic review.

## Results

### Corpus coverage and analytic outputs

The analysis produced two related outputs. First, a provision-level extraction dataset was developed, with each row representing a discrete, verifiable text unit from an instrument, accompanied by a source pointer and a verbatim excerpt. This process resulted in 104 extracted provision rows across the 15 instruments, of which 101 were retained for scored synthesis following quality-assurance screening. Second, the retained provision-level evidence was mapped to the benchmark framework’s 12 domains (D1–D12) and aggregated into an instrument-by-domain alignment matrix comprising 180 cells (15 instruments by 12 domains). Each cell indicates whether the instrument meets the minimum benchmark expectation for that domain, using the alignment score (A2 for complete alignment with the study-defined minimum, A1 for partial alignment, and A0 for no alignment). Delivery design was coded only for aligned cells (A ≥ 1) according to the decision rules specified in Table S1. Of the 180 instrument-by-domain cells, 34 were aligned, including five A2 and 29 A1 cells, while 146 cells were coded as not aligned. No cells were coded as indeterminate in the final scored dataset.

Unless otherwise stated, all coverage and alignment findings reported below refer exclusively to the 15 retrieved and scored instruments and should not be interpreted as evidence of jurisdiction-wide policy presence or absence.

### Provision volume and evidence concentration by domain

Within the retrieved and scored corpus, the 101 retained provision records were mapped to 11 of the 12 benchmark domains; no retained provision was mapped to D4 (food-marketing restrictions to protect children). Evidence was most concentrated in D8 (food environment measures beyond marketing) and D12 (monitoring, surveillance, evaluation, and reporting), each with 19 included provisions. D1 (child right to health and underlying determinants) accounted for 16 provisions, and D11 (multisector governance and policy coherence) for 14 provisions. Additional evidence was mapped to D9 (early life-course prevention; 11 provisions), D10 (obesity prevention and management within health services; 8 provisions), D5 (school food environment; 6 provisions), D2 (non-discrimination and child equity considerations; 3 provisions), D3 (business sector impacts and protection of policy integrity; 2 provisions), D7 (community/built environment for child physical activity; 2 provisions), and D6 (school physical activity environment; 1 provision) (Supporting Data).

The volume of provision extraction varied across instruments. The largest sets were recorded for Q11 (15 provisions), Q20 [14], Q9 [10], Q26 [10], and Q15 [9]. Mid-sized sets were observed for Q16 [7], Q5 [6], Q25 [6], Q3 [5], and Q14 [5], while the remaining instruments contributed smaller sets.

### Domain-level alignment results, reported by policy theme and instrument

#### System framing, equity, multisector governance, and monitoring (D1, D2, D11, D12)

For D1 (child’s right to health and underlying determinants), A2 was recorded for Q16, while partial alignment (A = 1) was recorded for Q9, Q11, Q20, and Q21. For D2 (non-discrimination and child equity considerations), partial alignment was recorded for Q9 and Q20, and no instrument was coded A2 in this domain. For D11 (multisector governance and policy coherence), A2 was recorded for Q11, with partial alignment for Q1, Q2, Q20, and Q21. For D12 (monitoring, surveillance, evaluation, and reporting), partial alignment was recorded for Q9, Q11, Q20, and Q26, with no instruments coded A2 (Table 4).

#### Business/regulatory environment and food-environment levers beyond marketing (D3, D8)

For D3 (business sector impacts and protection of policy integrity), partial alignment (A = 1) was recorded for Q3 only. For D8 (food environment measures beyond marketing), partial alignment was recorded for Q1, Q3, Q4, Q5, Q11, Q14, and Q15, with no instrument coded A2 in this domain (Table 4).

**Table 4:** Domain-level alignment summary

Domain	Domain label	Delivery specification among aligned cells: D2/D1/D0	Aligned instruments
D1	Child right to health and underlying determinants	0/2/3	A2: Q16; A1: Q9, Q11, Q20, Q21
D2	Non-discrimination & child equity considerations	0/0/2	A2: —; A1: Q9, Q20
D3	Business sector impacts and protection of policy integrity	1/0/0	A2: —; A1: Q3
D4	Food marketing restrictions to protect children (HFSS/“unhealthy”)	0/0/0	A2: —; A1: —
D5	School food environment	1/0/0	A2: —; A1: Q25
D6	School physical activity environment	0/1/0	A2: —; A1: Q26
D7	Community/built environment for child physical activity	0/0/2	A2: —; A1: Q9, Q11
D8	Food environment measures beyond marketing	2/2/3	A2: —; A1: Q1, Q3, Q4, Q5, Q11, Q14, Q15
D9	Early life-course prevention (maternal/infant/early childhood)	0/1/2	A2: Q14, Q15; A1: Q20
D10	Obesity prevention and management within health services	1/1/1	A2: Q23; A1: Q9, Q20
D11	Multisector governance and policy coherence (HiAP-type mechanisms)	3/2/0	A2: Q11; A1: Q1, Q2, Q20, Q21
D12	Monitoring, surveillance, evaluation, and reporting	3/1/0	A2: —; A1: Q9, Q11, Q20, Q26

Counts are instrument–domain cells across the 15 scored instruments. Delivery specification was coded only for A1- or A2-aligned cells. A2 indicates complete alignment with the study-defined domain minimum expectation; A1 indicates partial or limited alignment. Em dashes indicate no aligned instrument at that level. The absence of aligned instruments for a domain refers only to the 15 instruments in the retrieved and scored corpus and does not establish jurisdiction-wide policy absence

#### School-setting domains (D5, D6)

For D5 (school food environment), partial alignment was recorded for Q25 only, and for D6 (school physical activity environment), partial alignment was recorded for Q26 only. Because both instruments are explicitly time-bounded operational instruments, alignment in these domains is interpreted strictly within the school setting and the academic-year scope stated in the instrument text, without extrapolating to system-wide national coverage beyond what is documented.

#### Physical activity and supportive environments beyond schools (D7)

For D7 (community/built environment supporting child activity), partial alignment was recorded for Q9 and Q11 (Table 4).

#### Early life-course prevention and child/adolescent obesity services (D9, D10)

For D9 (early-life-course prevention), A2 was recorded for Q14 and Q15, and partial alignment for Q20. For D10 (child/adolescent obesity prevention and management services), A2 was recorded for Q23, with partial alignment for Q9 and Q20 (Table 4).

#### Domain with no aligned coverage within the scored corpus (D4)

Within the retrieved and scored corpus, D4 (food marketing restrictions to protect children) had no aligned instrument–domain cells; all 15 cells were coded as not aligned under the study-specific framework (Table 4). This finding concerns the scored evidence and does not establish the absence of relevant regulation elsewhere in Qatar’s policy landscape.

### Instrument-level alignment across benchmark domains

Across the 12-domain framework, the number of aligned domains per instrument (A ≥ 1) ranged from one to six (Table 4). The broadest profile was observed for Q20 (PHCC Corporate Strategic Plan), which aligned with six domains, followed by Q9 and Q11, each aligning with five domains. All remaining instruments aligned to one or two domains.

### Delivery plausibility for aligned cells

Delivery design was coded only for aligned cells (A ≥ 1) according to Table S1. Among the 34 aligned cells, delivery scores were distributed as follows: D2 = 11, D1 = 10, and D0 = 13. No indeterminate delivery codes were assigned (Table 4). This distribution suggests that some aligned commitments include codable delivery features such as specified responsibility, monitoring or reporting, accountability, or compliance mechanisms, while other aligned content remains primarily at the level of intent within the instrument text.

## Discussion

### Scope of interpretation

The Discussion interprets findings from the 15-instrument scored corpus against the benchmark domains. Statements about coverage, alignment, and delivery specificity apply only to content retrieved, extracted, and scored under the study’s evidence rules. Delivery-plausibility codes describe implementation-related features stated in the instrument text and do not demonstrate implementation, enforcement, effectiveness, service coverage, or population-level impact.

### Overall alignment pattern

At the instrument-by-domain level, the matrix comprises 180 cells (15 instruments by 12 domains). In the final scored matrix, 34 of 180 cells were aligned (5 A2 and 29 A1), 146 were coded as not aligned, and none were indeterminate. Of the 34 aligned cells, delivery plausibility was distributed as 11 D2, 10 D1, and 13 D0, showing that a substantial share of aligned content remains direction-setting or only partially operationalized at the instrument level.

### Food-environment action

Food-environment content in the scored corpus is concentrated in D8 (food-environment measures beyond marketing), with seven aligned instrument-domain cells across regulatory, fiscal, strategy, and dietary-guidance instruments. Extracted evidence is mainly drawn from regulatory provisions (food control/consumer protection), fiscal instruments (excise tax and its implementing regulation), and dietary guidance/strategy documents. In benchmark terms, these are plausible components of a wider food-environment approach, but they are generally framed as most effective when implemented as part of broader, multi-component policy packages that shape enabling environments, rather than as education-only responses [6, 7].

Within the retrieved and scored corpus, no provision met the minimum expectation for D4 (a child-focused HFSS marketing restriction package). This finding is relevant because the WHO’s 2010 Set of Recommendations calls for policies to reduce the impact of marketing on children of foods high in saturated fats, trans fats, free sugars, or salt, with monitoring and evaluation [27]. The 2023 WHO guideline further recommends comprehensive, mandatory measures to protect children from harmful food marketing, including coverage of digital media and attention to marketing techniques that increase persuasive power [43]. A benchmark-consistent child-focused marketing restriction package was therefore not demonstrated under the applied coding rules. This is a corpus-bounded finding and does not establish that relevant legal, regulatory, or administrative measures are absent elsewhere in Qatar’s policy landscape. Regional reporting indicates that GCC countries have adopted differing combinations of healthy-diet measures, including fiscal policies, reformulation initiatives, school-related nutrition measures, and selected actions addressing food marketing, although their scope and status vary across countries [31]. These measures differ in scope and were not assessed using the provision-level framework applied to Qatar. The absence of aligned D4 evidence in the scored Qatar corpus should therefore be viewed in the context of a broader regional variation in the scope and status of child-focused food-marketing regulation. Regional proposals and voluntary measures may also fall outside the scored corpus, including draft or voluntary regional measures addressing food marketing [44]. Such draft, voluntary, or non-governmental materials were not scored because the study was restricted to authoritative governmental primary instruments; they would not, by themselves, satisfy the D4 minimum for a mandatory child-focused restriction package. Their exclusion reflects the corpus boundary rather than a judgment that such materials are irrelevant.

### School policy results: partial coverage (D5–D6)

Q25 (school-canteen supplier qualification specifications) and Q26 (school fitness evaluation program and standards) provide extractable provisions specific to school settings. Under the applied extraction rules, these instruments contributed one partial alignment in D5 (school food environment) and one partial alignment in D6 (school physical-activity environment), with no A2 alignment in either domain. The scored corpus, therefore, contains reviewable school-setting content, although its coverage remains limited and time-bounded. Recent WHO guidance further underscores the relevance of durable school food standards, nutrition rules, and implementation arrangements, although it was not part of the pre-specified benchmark corpus [45].

Accordingly, school-setting alignment in the scored corpus remains partial and dependent on two time-bounded instruments. This finding does not establish that broader, more recent, or continuing school policies are absent from Qatar’s wider policy landscape.

### Equity and commercial determinants

Two benchmark-derived domains, foundational to the framework, demonstrate only limited coverage in the scored corpus. Specifically, D2 (equity/non-discrimination) exhibits two partial alignments and no A2 alignment, while D3 (business-sector impacts and policy integrity) shows one partial alignment and no A2 alignment. In contrast, rights-based benchmarks treat non-discrimination as a core obligation (CRC, Article 2) and establish the protection of children’s rights from adverse business impacts as a state duty (CRC General Comment No. 16), in addition to broader obligations to realize the child’s right to health by addressing inequities (CRC General Comment No. 15) [3–5]. Public health scholarship similarly identifies commercial determinants and the practices of “unhealthy commodity” industries as significant upstream influences on diet-related risk and non-communicable disease prevention efforts [5, 23]. The matrix thus supports a narrow inference: within this scored obesity-relevant set, explicit and extractable provisions that meet minimum expectations for equity-sensitive design and systematic management of business-sector impacts or policy integrity are limited and do not meet the A2 threshold. This finding is analytically distinct from broader claims regarding Qatar’s overall legal framework and reflects only what is evidenced in this scored set under the applied extraction rules.

### Interpreting alignment and delivery specificity

A central contribution of this framework is the distinction between A-codes (indicating whether a minimum expectation is met) and D-codes (indicating whether aligned content is accompanied by delivery or implementation features). This distinction is important because two corpora may show similar alignment counts yet differ in the specificity of their textual delivery. Implementation science frameworks similarly emphasize that adoption and routinization depend on feasibility, fidelity, accountability, and sustainment, rather than solely on the existence of policy statements [41].

### Gap typology and policy-development considerations

Applying the benchmark decision rules and minimum expectations outlined in Supporting Table S1 (A-code and D-code logic), the gap register distinguishes between two analytically distinct gap types in the scored Qatar corpus: coverage gaps, where benchmark domains lack *extractable*,* scorable alignment*, and design-quality gaps, where alignment exists but the *implementation architecture evident in the text* is limited or inconsistent. This distinction is significant because a coverage gap indicates a missing benchmark pillar *within the scored corpus*, while a design-quality gap indicates that a pillar is present but does not meet the higher textual-specification criteria under the study’s reviewability logic.

### Coverage gaps and limited coverage in benchmark-salient domains

Analysis of the provision-level dataset and instrument–domain summaries identified one zero-alignment domain within the scored corpus: D4 (food marketing restrictions to protect children), with no retained provisions and no instruments demonstrating scorable alignment in this domain. This classification is particularly salient because the WHO’s marketing recommendations and the 2023 guideline identify reductions in children’s exposure to unhealthy food marketing as a core upstream protection, emphasizing that effectiveness relies on explicit policy scope, definitional clarity, implementation considerations, and accountability features [27, 43]. This classification indicates that the retrieved and scored instruments did not demonstrate the benchmark minimum for D4; it should not be interpreted as confirmation that Qatar has no food-marketing regulation.

Where alignment is present, the scored corpus demonstrates a consistent design-quality pattern: most aligned provisions are partial matches, and many serve as direction-setting rather than highly specified at the text-unit level. In the extraction set (*n* = 101 provisions), A1 codes predominate (81/101) compared to A2 (20/101), and delivery plausibility is distributed as D0 (43/101), D1 (23/101), and D2 (35/101). A key design-quality indicator is the alignment–delivery disconnect: several A2 provisions were coded as D0, suggesting that strong benchmark correspondence sometimes appeared as normative or guidance-oriented statements without embedded implementation features in the same extractable unit. This indicates an operational-specification gap, suggesting that strengthening could involve linking A2-aligned intent to more explicit delivery specifications, such as ownership, cadence, measurable requirements, and reviewable accountability, rather than merely increasing the volume of aligned text.

### Domain-level design-quality patterns

Cross-cutting governance attributes in the provision-level data further clarify areas where design quality is limited. Accountability is often unspecified at the provision level: the accountability field is populated in 94 out of 101 provisions, but 51 explicitly record “not specified.” Monitoring and reporting are present in 59 provisions yet are predominantly coded as unclear [34] or internal [20], with only a small number referencing concrete key performance indicators or delivery-unit structures. Enforcement design is least visible, with explicit sanctions appearing in only 5 provisions (all within binding legal instruments), and no conflict-of-interest safeguards were identified in the 26 records for which the COI field was coded. This pattern indicates that many aligned excerpts support benchmark correspondence through intent or internal management framing, but do not consistently provide externally reviewable accountability mechanisms or integrity safeguards. These features are repeatedly emphasized in the WHO governance and integrity guidance for nutrition policy [46]. Table 5 summarises the coverage and design-quality gaps identified within the retrieved and scored corpus, together with the supporting evidence and potential policy-development directions.

**Table 5:** Gap typology and actionable gaps in the scored policy corpus (coverage vs. design-quality gaps)

Domain/gap	Gap type	Evidence in scored corpus	Action direction
**D2 – Equity / vulnerable groups**	Design-quality	3 provisions, 2 instruments (Q9, Q20); A2 = 0.	Make equity explicit and reviewable (named groups, access/affordability) and add equity indicators and reporting.
**D3 – Commercial determinants / integrity**	Design-quality	2 provisions, 1 instrument (Q3); A2 = 0.	Add integrity safeguards (transparent engagement, disclosure/recusal, participation boundaries) linked to obesity-relevant policy processes.
**D4 – HFSS marketing restrictions**	Coverage	0 provisions, 0 instruments aligned for D4 in this set.	Develop (or include if outside this set) a child-focused HFSS marketing package with scope/definitions and monitoring and enforcement.
**D5 – School food environment**	Design-quality	6 provisions, but 1 instrument only (Q25); A2 = 0.	Move toward durable school food standards with compliance expectations and routine monitoring/reporting.
**D6 – School PA environment**	Design-quality	1 provision, 1 instrument (Q26); A2 = 0.	Specify minimum PA standards, responsible entity, and monitoring cadence.
**D7 – Community/built environment for child PA**	Design-quality	2 provisions, 2 instruments (Q9, Q11); A2 = 0.	Specify child-relevant built-environment, transport, and recreation mechanisms with responsible actors, implementation pathways, and monitoring indicators.
**D8 – Food environment beyond marketing**	Design-quality	19 provisions, 7 instruments (Q1, Q3, Q4, Q5, Q11, Q14, Q15); A2 = 0.	Consolidate into a coherent D8 bundle with explicit monitoring and ensure it complements D4 and school measures.
**GC-01 – COI safeguards**	Design-quality	COI field coded in 26/101; all “none.”	Add COI rules (disclosure, recusal, participation limits) and transparency requirements.
**GC-02 – Monitoring and evaluation**	Design-quality	Monitoring coded in 59/101; mainly Unclear (34) or Internal (20); 5 structured.	Standardise indicators, cadence, and responsibility, and add externally reviewable reporting where feasible.

All instruments were analyzed using publicly available English-language versions issued or hosted by official governmental or institutional sources. No researcher-generated translation was undertaken

*Abbreviations*: *D1–D12 * benchmark domains, *HFSS*  foods high in saturated fats, trans-fatty acids, free sugars and/or salt, *PA*  physical activity, *COI*  conflict of interest. Coverage gaps = no extractable alignment in the scored corpus; Design-quality gaps = alignment exists but is thin/partial or not highly specified under the Table S1 decision rules. GC-01/GC-02 are cross-cutting governance attributes (not domains): GC-01 refers to COI safeguards, and GC-02 refers to monitoring/reporting design

**Fig. 2:**
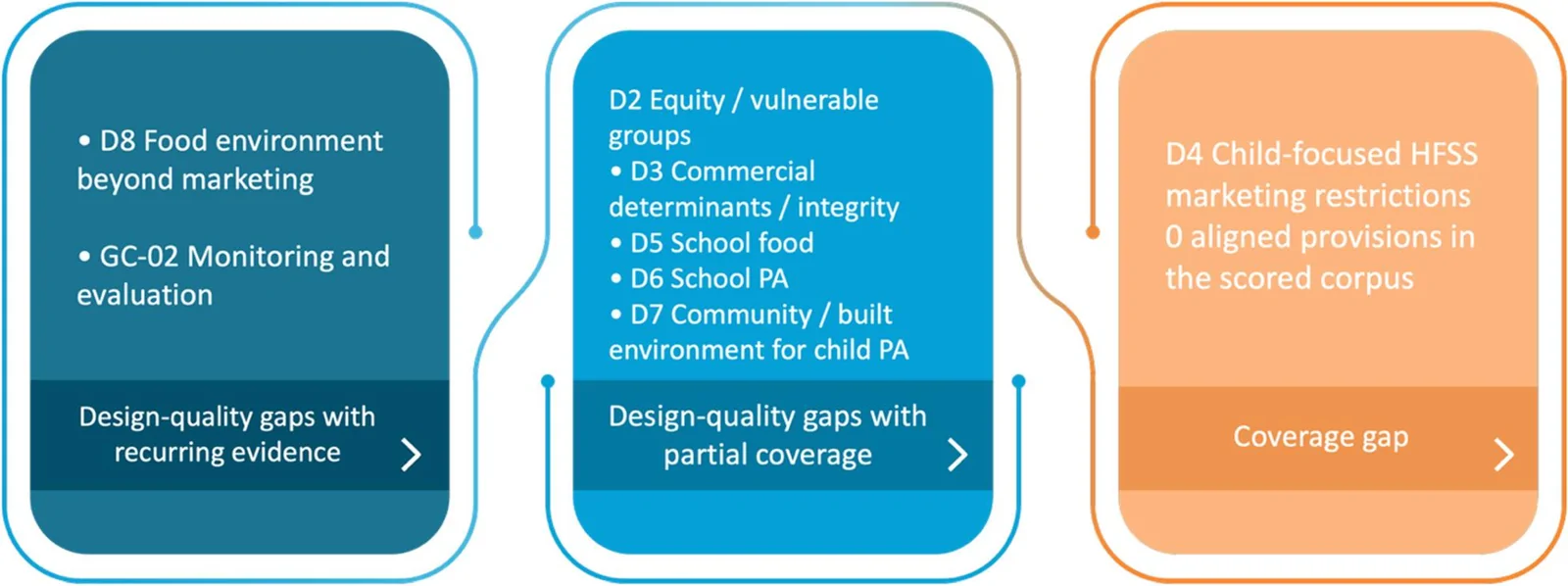
Gap profile of the scored Qatar policy corpus. The figure summarizes coverage and design-quality findings within the retrieved and scored instruments, including the absence of aligned D4 evidence in this corpus and cross-cutting governance gaps related to COI safeguards and monitoring/reporting design. It does not represent a jurisdiction-wide assessment of the absence of policy

Figure 2 summarises the gap types identified in the scored Qatar policy corpus. It distinguishes the main coverage gap, D4 child-focused HFSS marketing restrictions, from design-quality gaps where evidence was present but limited, partial, time-bounded, or limited in textual delivery specification. It also highlights cross-cutting governance gaps related to COI safeguards and monitoring/reporting design.

### Policy implications arising from the scored corpus

Within the scored corpus, the most frequently documented areas were strategic direction, health-service guidance, and non-marketing food-environment measures. However, the corpus showed less comprehensive coverage in areas where benchmark frameworks emphasize upstream, child-specific protections and sustained, settings-based prevention. These findings suggest that future verification and policy development could focus on complementing downstream guidance and monitoring with a limited set of more explicitly specified upstream instruments, rather than increasing documentation alone.

A priority is to verify whether a current, comprehensive, child-focused food-marketing instrument exists outside the scored corpus. If no such instrument is identified, future policy development could consider explicit scope, definitions, media and setting coverage, monitoring, accountability, and enforcement provisions consistent with the benchmark expectations. International guidance consistently identifies marketing exposure as a critical upstream determinant, emphasizing that effective protection requires mandatory coverage across all media and settings, clear nutrient or food classification, a defined age range for children, and enforceable monitoring and remedies [27, 29, 47]. If such a gap is confirmed, an appropriately designed instrument could strengthen the commercial determinants pillar and improve alignment with benchmark standards across food-environment domains.

If further retrieval confirms that current school provisions remain time-bound, consideration could be given to consolidating them into a durable, comprehensive prevention package. Benchmark frameworks identify schools as settings for integrated prevention, including food standards and procurement, restrictions on unhealthy options, support for physical activity, and monitoring and accountability [8, 26, 45]. Where gaps are confirmed, future instruments could establish durable standards, clear compliance expectations, and routine reporting or audit cycles [8].

Within the scored corpus, equity and rights protections are not consistently articulated in scorable, obesity-relevant provisions, limiting the ability to demonstrate, through transparent evidence, that prevention efforts are designed to protect children at higher structural risk. When relevant instruments are revised or developed, equity-by-design features could be made explicit, including targeted protections for high-risk groups, universal-access design, affordability safeguards, and measurable equity indicators for monitoring. This would make equity a reviewable policy commitment aligned with rights-based benchmark standards [4, 8].

The governance findings from the scored corpus suggest that future reviews or revisions of relevant instruments could prioritize clearer ownership, delivery specifications, and accountability mechanisms. Implementation science literature emphasizes the value of clearly specifying roles, workflows, feedback loops, and performance mechanisms, rather than relying solely on policy intent [39, 41]. Therefore, future policy revisions and new instruments could more explicitly state delivery-related features, including designated responsible actors, timelines, delivery mechanisms, resource expectations, and clearly defined monitoring and escalation pathways.

For example, the Q23 direction-setting provision on the prevention, diagnosis, and management of obesity in children was coded A2/D0 because it stated the substantive objective but lacked delivery-related features. A hypothetical linked provision that assigned a responsible service entity, defined a standardized screening and referral pathway, and required annual reporting of coverage indicators would include multiple independent delivery features and therefore meet the D2 textual specification threshold. This example is illustrative and does not indicate that these arrangements currently exist.

No explicit conflict-of-interest safeguards were identified in the scored text units where this attribute was assessed. This indicates a corpus-level specification gap, not evidence that such safeguards are absent from all relevant governance arrangements. WHO has emphasized the need for systematic approaches to prevent and manage conflicts of interest in nutrition policy, particularly when commercial actors may have incentives that conflict with public health goals [46]. Future instruments could specify conflict-of-interest governance, including disclosure requirements, participation rules, recusal and decision protocols, and transparency in stakeholder engagement, so that integrity protections are explicit and reviewable.

Beyond Qatar, the structured document retrieval, provision-level extraction, and dual alignment–delivery coding workflow offer a potentially replicable diagnostic framework for the wider GCC. Future research could apply a harmonized benchmark matrix across GCC member states to generate comparable national policy profiles and then examine regional similarities and differences in policy coverage and in the specificity of textual delivery, monitoring, and accountability. Such an application should retain country-specific legal and institutional interpretations rather than assuming that policies or legal terminology are directly equivalent across jurisdictions.

### Evaluation of governance design quality within the scored evidence

Governance design quality is assessed using the study’s delivery plausibility code (D-code) alongside provision-level design fields from the results pack, including specificity level, policy lever type, provision type, and the child-specificity flag. Analysis of 101 provision rows reveals a clear textual delivery-specificity gradient: 43 provisions are primarily direction-setting (D0), 23 exhibit partial delivery features (D1), and 35 contain two or more codable delivery-related features (D2). These findings indicate that the scored corpus contains a substantial number of provisions with coded delivery-related features. Nevertheless, a considerable portion of alignment relies on text that signals intent or guidance rather than specifying multiple delivery-related features, such as explicit operational requirements, compliance logic, or system routines [39, 41].

Provisions with two or more coded delivery-related features are concentrated within policy lever types rather than evenly distributed across the policy package. In the results pack, all monitoring and management provisions are classified as D2 (11 out of 11), as are all school food-environment provisions (6 out of 6). Fiscal provisions are predominantly D2 (7 out of 9). In contrast, informational provisions lack D2 content (0 out of 26) and are almost entirely D0 (24 out of 26), while governance provisions are mixed (D0 = 16, D1 = 9, D2 = 10). These patterns indicate that the strongest concentration of textual delivery features in the scored corpus is evident in policies articulated through management systems, operational school requirements, and fiscal instruments. By contrast, much of the aligned narrative content, particularly strategic and informational provisions, does not meet the D2 textual specification threshold at the provision level used for benchmarking.

Child-specificity coding reveals a design trade-off relevant to the explicitness of child protection. Child-explicit provisions (CE = 36) are primarily direction-setting (D0 = 24 out of 36), while D2-coded provisions (D2 = 35) are more often child-implicit (CI = 26 out of 35) than child-explicit (CE = 9 out of 35). This pattern does not suggest that delivery is not child-focused, but rather that many D2-coded levers are articulated in general terms. For future instrument design, the most operational provisions, particularly those already D2-coded, such as monitoring and management and school food-environment requirements, could explicitly incorporate child-protective objectives, thresholds, and performance expectations, rather than relying on inferred child relevance. The most defensible interpretation from the scored evidence is that the scored corpus contains pockets of higher textual delivery specification (notably monitoring and management, school food operational requirements, and fiscal instruments), but that many of the strongest alignment statements remain limited in delivery specification at the provision level.

### Limitations

These conclusions should be interpreted within the study’s design and corpus boundaries. The single-country design limits the generalizability of the findings to other GCC and MENA jurisdictions with different legal systems, institutional arrangements, and policy-publication practices. The scored corpus represents a limited, text-retrievable subset of Qatar policy instruments identified during the study period, derived from publicly accessible primary documents and predefined extraction rules. The document search was limited to publicly accessible, retrievable materials. Exact repository-specific query strings, source-specific search dates, the language used for each individual query, and total hit counts were not logged prospectively. As a result, the findings describe what is demonstrable within the scored set and should not be generalized to Qatar’s full policy landscape without additional primary-text retrieval and corpus expansion. In particular, the absence of extractable evidence for certain benchmark packages, such as child-focused marketing restrictions, in the scored set should be read as a corpus-bounded finding, not as proof of jurisdiction-level absence.

The A- and D-codes are study-specific ordinal classifications developed to organize provision-level evidence and have not undergone external validation. Coding was conducted by a single researcher; no blinded independent review or inter-rater reliability assessment was performed. Although explicit decision rules, primary-text excerpts, location pointers, and quality-assurance records were used to support consistency and transparency, the selected category definitions and thresholds may have influenced classification and should be examined in future independent applications. The analysis did not include stakeholder perspectives from policymakers, schools, healthcare providers, civil society organizations, children, or families; therefore, it cannot determine how the identified instruments are interpreted, coordinated, implemented, or experienced in practice. Finally, because the analysis benchmarks policy text for alignment and delivery or accountability features, it does not evaluate implementation fidelity, enforcement in practice, resourcing, coverage, or population-level effects. Therefore, the conclusions pertain to documented policy architecture rather than real-world impact.

## Conclusions

This benchmarking analysis establishes an auditable, text-based baseline of demonstrable content from a set of Qatar policy instruments, assessed against child-rights and WHO benchmarks. The primary finding is not a lack of policy in Qatar; rather, the policy content within the scored documents is unevenly distributed. The analysis identifies stronger coverage in areas such as strategic intent and guidance, including nutrition and physical activity guidance, governance and monitoring, and clinical guidance and strategic commitments relevant to child and adolescent weight management.

Two notable corpus-level gaps emerge from the retrieved texts under the applied extraction rules. First, the scored set lacks sufficient evidence to meet minimum expectations for a child-focused food marketing restriction package. Second, although school-setting content is present, it remains limited and does not constitute a benchmark-consistent school policy package across documents. These findings are limited to the analyzed corpus and indicate only what was not demonstrated in the scored set, rather than what exists in Qatar as a whole.

Priority next steps include expanding and confirming the corpus through primary-text retrieval, particularly for marketing restrictions and school policy packages, and considering strengthening textual delivery and accountability features where relevant instruments are revised or developed. This approach enables iterative updates and supports more implementation-oriented assessments while avoiding overstatement. The framework also provides a methodological basis for harmonized application across GCC member states, which could support future regional comparative analysis while preserving country-specific legal and policy contexts.

## Supplementary Information

Supplementary Material 1.

## Data Availability

The domain framework, coding rules, and source-provenance summary are provided in the supplementary information.

## Abbreviations

A-code: Alignment code
A0: No alignment
A1: Partial or limited alignment
A2: Complete alignment with the study-defined minimum
A9: Indeterminate alignment
BMI: Body Mass Index
CFIR: Consolidated Framework for Implementation Research
COI: Conflict of Interest
CRC: Convention on the Rights of the Child
D-code: Delivery plausibility code
D0: No codable delivery features
D1: At least one codable delivery feature
D2: Two or more codable delivery features
D9: Indeterminate delivery plausibility
DPAS: Global Strategy on Diet, Physical Activity and Health
ECHO: Commission on Ending Childhood Obesity
EMRO: WHO Regional Office for the Eastern Mediterranean
GAPPA: Global Action Plan on Physical Activity
GCC: Gulf Cooperation Council
GC-01: Cross-cutting governance attribute for conflict-of-interest safeguards
GC-02: Cross-cutting governance attribute for monitoring/reporting design
HFSS: Foods high in saturated fats, trans-fatty acids, free sugars and/or salt
HiAP: Health in All Policies
HMC: Hamad Medical Corporation
MENA: Middle East and North Africa
MOEHE: Ministry of Education and Higher Education
NCD: Noncommunicable disease
NDS3: Third National Development Strategy 2024–2030
PA: Physical activity
PHCC: Primary Health Care Corporation
PRISMA: Preferred Reporting Items for Systematic Reviews and Meta-Analyses
QA: Quality assurance
QNPAG: Qatar National Physical Activity Guidelines
UN: United Nations
UNICEF: United Nations Children’s Fund
WHA: World Health Assembly
WHO: World Health Organization
