# Supplementary material for: Childhood obesity governance in Qatar: a policy and legal benchmarking analysis against international child rights and WHO frameworks
Source: BMC Public Health. 2026 Aug 4;26:3138. doi: 10.1186/s12889-026-28785-0 (PMC13721363; doi:10.1186/s12889-026-28785-0)
Supplement: Supplementary file 1 [file 12889_2026_28785_MOESM1_ESM.docx]

**Supporting Information**

**Childhood Obesity Governance in Qatar: A Policy and Legal Benchmarking Analysis Against International Child Rights and WHO Frameworks**

**Mariam Alisi¹˒²**

¹ Department of Social & Policy Sciences, University of Bath, Claverton Down, Bath BA2 7AY, United Kingdom.

² College of General Education, University of Doha for Science and Technology, Al Tarafa, Duhail North, P.O. Box 24449, Doha, Qatar.

Correspondence: mariam.alisi@udst.edu.qa

Table S1. Benchmark-derived domains, operational definitions, and study-specific coding rules.

| Domain | Benchmark-derived domain | Operational meaning  (minimum expectation) | Primary anchor instruments | Alignment classification (A-code) |
| --- | --- | --- | --- | --- |
| D1 | Child right to health and underlying determinants | Explicit child-health protection linked to underlying determinants relevant to diet, physical activity, nutrition, or healthy development. | CRC Art. 24; CRC GC15; DPAS; ECHO | General A-rule. |
| D2 | Non-discrimination and child equity considerations | Explicit, reviewable equity or non-discrimination features affecting children’s access to prevention or health-supportive environments. | CRC Arts. 2–3; CRC GC15 | General A-rule. |
| D3 | Business sector impacts and protection of policy integrity | Duties to regulate or oversee business impacts on children’s health or rights, including harmful marketing, and/or explicit policy-integrity or conflict-of-interest safeguards. | CRC GC16; HiAP; WHO 2010 and 2023 marketing instruments | General A-rule. Conflict-of-interest safeguards are coded separately and are not required for A2 unless they form part of the operative domain minimum. |
| D4 | Food marketing restrictions to protect children (HFSS/‘unhealthy’ products) | Measures reducing children’s exposure to, and the persuasive power of, unhealthy-food marketing, with codable scope, media/settings, techniques, monitoring, or enforcement features where stated. | WHO 2010 recommendations; WHO 2023 guideline; CRC GC16 | A2 when a child-focused restriction package meets the study-defined minimum; A1 when relevant restrictions are limited or incomplete. |
| D5 | School food environment | School or childcare food standards, restrictions, procurement requirements, or availability rules, including compliance features where stated. | ECHO; DPAS | General A-rule. |
| D6 | School physical activity environment | School physical-activity or physical-education requirements, standards, opportunities, or supportive-setting provisions. | DPAS; GAPPA; ECHO | General A-rule. |
| D7 | Community/built environment for child physical activity | Supports for active living through built environment, transport, recreation, or community settings, where child relevance is explicit or defensible. | GAPPA; DPAS | General A-rule. |
| D8 | Food environment measures beyond marketing | Upstream food-environment levers beyond marketing, including standards, procurement or public-sector measures, fiscal measures, and other enabling-environment provisions. | DPAS; WHO Global NCD Action Plan; WHO Obesity Acceleration Plan; EMRO framework (context) | General A-rule; A2 does not require multiple different policy levers. |
| D9 | Early life-course prevention (maternal/infant/early childhood) | Pregnancy, infancy, or early-childhood nutrition, healthy growth, breastfeeding-related support, or other early-life prevention measures. | CRC Art. 24; CRC GC15; ECHO | General A-rule. |
| D10 | Obesity prevention and management within health services | Child- or adolescent-relevant commitments concerning obesity prevention support, diagnosis, management, or treatment, only to the level stated. | ECHO; WHO Obesity Acceleration Plan; EMRO framework (context) | General A-rule. |
| D11 | Multisector governance and policy coherence (HiAP-type mechanisms) | Mandates, assigned roles, coordination mechanisms, and whole-of-government or whole-of-society governance structures. | HiAP; WHO NCD Action Plan; WHO NCD Roadmap; WHO Obesity Acceleration Plan | General A-rule. |
| D12 | Monitoring, surveillance, evaluation, and reporting | Surveillance, monitoring, evaluation, reporting, or accountability commitments that make policy content reviewable. | WHO NCD Action Plan; WHO NCD Roadmap; CRC GC15; HiAP; EMRO framework (context) | General A-rule. Ownership and reporting cadence strengthen the D-code and are not required for A2 unless part of the domain minimum. |

**Coding rules:** The general A-rule is A2 = complete alignment with the study-defined minimum expectation; A1 = relevant but incomplete alignment; A0 = no qualifying aligned evidence; and A9 = indeterminate from the available text. A2 does not indicate comprehensive policy adequacy, legal compliance, implementation, or national performance.

**D-code rules:** D is coded only when A1 or A2 is present. D0 = no codable delivery-related feature; D1 = one distinct feature; D2 = two or more distinct features; and D9 = indeterminate. Codable features include assigned responsibility, implementation or compliance mechanisms, monitoring or reporting, enforcement, and accountability, where explicitly stated. Features are not weighted, and D2 indicates greater textual specification of delivery rather than demonstrated implementation capacity, fidelity, effectiveness, or performance.

Abbreviations: CRC = UN Convention on the Rights of the Child; GC15/GC16 = General Comment No. 15 / No. 16; WHO = World Health Organization; ECHO = Commission on Ending Childhood Obesity; DPAS = Global Strategy on Diet, Physical Activity and Health; GAPPA = Global Action Plan on Physical Activity; NCD = noncommunicable disease; EMRO = WHO Regional Office for the Eastern Mediterranean; HiAP = Health in All Policies; HFSS = foods high in saturated fats, trans-fatty acids, free sugars and/or salt.

Table S2. Source provenance, broad eligibility/search concepts, and screening summary for the scored Qatar corpus.

| Official source or issuing body | Broad eligibility/search concepts  (summary) | Included instrument IDs | Number included | Archived source files |
| --- | --- | --- | --- | --- |
| Al Meezan Qatar Legal Portal (almeezan.qa) | food regulation and safety; consumer protection; food advertising; public health | Q1–Q3 | 3 | Q1.pdf; Q2.pdf; Q3.pdf |
| General Tax Authority (gta.gov.qa) | excise taxation; sweetened beverages; carbonated beverages; energy drinks | Q4–Q5 | 2 | Q4.pdf; Q5.pdf |
| National Planning Council (npc.qa) | national development; population health; nutrition; physical activity; wellbeing; monitoring | Q9 | 1 | Q9.pdf |
| Ministry of Public Health and official platforms (moph.gov.qa; hiap.moph.gov.qa) | child health; childhood obesity; health strategy; dietary guidance; maternal and child nutrition; obesity management | Q11, Q14, Q15, Q23 | 4 | Q11.pdf; Q14.pdf; Q15.pdf; Q23.pdf |
| Aspetar (aspetar.com) | physical-activity guidance; children and adolescents; schools; active living | Q16 | 1 | Q16.pdf |
| Primary Health Care Corporation (phcc.gov.qa) | primary-care strategy; child health; obesity; nutrition; health promotion; prevention; monitoring | Q20 | 1 | Q20.pdf |
| Hamad Medical Corporation (hamad.qa) | healthcare strategy; child and adolescent services; obesity; prevention; governance | Q21 | 1 | Q21.pdf |
| Ministry of Education and Higher Education (edu.gov.qa and official document host) | school food; school canteens; school nutrition; student health; physical education; physical fitness | Q25–Q26 | 2 | Q25.pdf; Q26.pdf |
| Total |  | **15 scored instruments** | **15** |  |

**Search and screening note:** The corpus was finalized on 6 February 2026.

All 15 scored instruments were analyzed using publicly available English-language versions. The original inventory contained 26 candidate instruments: 15 met the inclusion criteria and were scored; the remaining 11 did not meet one or more criteria.
